# Short‐term clinical observations of belimumab in the treatment of recently diagnosed systemic lupus erythematosus

**DOI:** 10.1002/iid3.716

**Published:** 2022-10-11

**Authors:** Chunye Wu, Baoqi Gong

**Affiliations:** ^1^ Department of Immunology The Tianjin First Central Hospital Tianjin China

**Keywords:** belimumab, high disease activity, recently diagnosed systemic lupus erythematosus, therapeutic effect

## Abstract

**Objective:**

To explore the therapeutic effectiveness and safety of belimumab in the treatment of recently diagnosed systemic lupus erythematosus (SLE).

**Methods:**

Between January 2019 and February 2022, a total of 30 patients who had been recently diagnosed with SLE were selected for 6 months of belimumab treatment at the Department of Rheumatology and Immunology, Tianjin First Central Hospital. Laboratory test results and related adverse reactions were recorded at baseline and after treatment.

**Results:**

Participants' white blood cell counts and complement 3, complement 4, and hemoglobin levels were higher after treatment than at baseline. Participants' immunoglobulin G and immunoglobulin M levels, SLE Disease Activity Index 2000 scores, glucocorticoid doses, erythrocyte sedimentation rates, and serum albumin/globulin ratios were lower after treatment. These differences were all statistically significant (*p* < .05).

**Conclusion:**

Belimumab was safe and effective in patients recently diagnosed with SLE and might help to reduce the use of glucocorticoids and to improve anemia with few adverse reactions. Belimumab might be applied in the treatment of patients recently diagnosed with SLE with high disease activity.

## Graphical abstract

This study aims to explore the therapeutic effectiveness and safety of belimumab in the treatment of recently diagnosed systemic lupus erythematosus (SLE). Belimumab was safe and effective in patients recently diagnosed with SLE and might help to reduce the use of glucocorticoids and to improve anemia with few adverse reactions. Belimumab might be applied in the treatment of patients recently diagnosed with SLE with high disease activity.

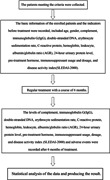

## 1 INTRODUCTION

Systemic lupus erythematosus (SLE) is an autoimmune disease that is characterized by multisystem and organ involvement, the presence of a large number of autoantibodies, and recurrent relapses and remissions. If untreated, it may cause irreversible damage to the affected organs and can even lead to death.^1^ The current standard treatment for SLE is glucocorticoids and immunosuppressants, but long‐term use of these treatments has many side effects, and some patients fail to respond well to or are intolerant to these standard treatments.^2^ The etiology of SLE is not yet fully understood, though abnormal B‐cell activation is a key component of the pathogenesis. Belimumab is a human‐derived immunoglobulin G (IgG) lλ monoclonal antibody that specifically targets soluble human B lymphocyte‐stimulating factor.^3^ Multiple studies have confirmed that belimumab may be applied in the treatment of SLE,^4^ and some results suggest that it should be adopted as early as possible to maximize the therapeutic effects.^5^ The current study aimed to investigate the effectiveness and safety of belimumab treatment in Chinese patients recently diagnosed with SLE.

## 2 MATERIAL AND METHODS

### 2.1 Participants

A total of 30 patients (2 males and 28 females) who had recently been diagnosed with SLE were selected to participate in the study. These patients were between 16 and 55 years old, with an average age of 30.86 ± 12.06 years. Their disease durations ranged from 1 to 10 months, with an average duration of 8.71 ± 4.55 months. Of the 30 participants, 19 experienced hematologic involvement, 12 renal involvement, 10 cutaneous involvement, and 4 serositis.

The study took place between January 2019 and February 2022 at the Tianjin First Central Hospital. The inclusion criterium was a diagnosis of SLE following the 2019 European League Against Rheumatism/American College of Rheumatology criteria.^6^ The exclusion criteria were severe and active central nervous system involvement, severe active lupus nephritis (LN), or hepatitis B or C virus infection. Enrolled participants signed an informed consent form, and the study was approved by the hospital's medical ethics committee. Severe acute LN was defined as 24‐h urine protein quantification of >6 g or equivalent urine protein‐to‐creatinine ratio, serum creatinine of >2.5 mg/dl, severe active nephritis requiring urgent treatment, required hemodialysis within 90 days before Day 0, or high‐dose prednisone (>100 mg/day).

### 2.2 Therapeutic regimen

The standard treatment regimen was determined by each participant's clinician, and all patients were treated with glucocorticoids plus immunosuppressants. All participants were treated with hydroxychloroquine. In two cases, this was the sole therapeutic. It was combined with tacrolimus in eight cases, cyclophosphamide in three cases, mycophenolate mofetil in ten cases, and cyclosporine in seven cases. For the study protocol, 10 mg/kg of belimumab was administered intravenously on days 1 (the baseline), 15, and 29, and once every 28 days afterward. The patients' conditions and any adverse reactions were assessed every 4 weeks. If there was no improvement in disease control after 6 months of treatment, discontinuation of the belimumab therapy was considered. Participants' doses of glucocorticoids and immunosuppressants were adjusted by their doctors as appropriate, and the relevant immunological parameters were tested regularly.

### 2.3 Observation indicators and detection methods

Testing was conducted at baseline and 24 weeks after the protocol treatment. The laboratory tests conducted for the study included complement 3 (C3), complement 4 (C4), IgG, immunoglobulin M (IgM), double‐stranded DNA (dsDNA), erythrocyte sedimentation rate (ESR), C‐reactive protein, hemoglobin (Hb), white blood cell count (WBC), platelet count (PLT), serum albumin/globulin ratio (A/G ratio), and the 24‐h urine protein test. At the testing timepoints, participants' doses of glucocorticoids were recorded, and the SLE Disease Activity Index 2000 (SLEDAI‐2 K) was completed. Any adverse reactions were observed during the treatments.

The total clinical therapeutic effectiveness was determined from the comparison of after‐treatment data with the baseline. In cases categorized as *obvious effect*, the clinical symptoms had disappeared completely after treatment. Immune globulin and complement returned to normal. In those categorized as *effect*, symptoms were effectively relieved (including the levels of complement and anti‐ds‐DNA), and immunological indicators improved by 1/3 or more than the initial results. In cases considered *no effect*, the clinical symptoms did not improve or even deteriorated after treatment, and the serological indicators either did not improve or worsened. The total effectiveness for the study was calculated as ([the number of cases with obvious effects + the number of cases with effects]/the total number of cases) × 100%.

Observed adverse reactions to treatment were infusion and hypersensitivity reactions, headache, nausea, vomiting, skin rash, infection, and leukopenia.

### 2.4 Statistical methods

The SPSS 25.0 statistical software was used for data processing. Measurement data that satisfied the normal distribution were expressed as mean ± standard deviation (or $\bar{x}$ ± *s*), and the data collected at baseline and after treatment were compared using the paired samples *t* test. Measurement data that did not satisfy the normal distribution were expressed as median (25th percentile to 75th percentile). The Wilcoxon rank‐sum test for paired samples was used for these comparisons. The countable data were expressed as the number of cases and percentages. Statistical significance was set to *p* < .05.

## 3 RESULTS

### 3.1 Immune indicators at baseline and after treatment

Of the 19 patients with blood involvement, 10 patients had leukopenia. The leukocyte counts of these 15 patients ranged between 3 ×10^9^ and 3.9 × 10^9^/L, with a median of 3.6 × 10^9^/L, and went back to normal after treatment. Four patients were anemic, and the Hb levels were 105, 108, 111, and 112 g/L before treatment. The Hb returned to normal after treatment. After treatment, C3, C4, WBC, PLT, and Hb levels and the A/G ratio were higher than they were at baseline, as shown in Table 1.

**Table 1:** The changes of indicators before and after treatment in the patients

	*n*	WBC (×10^9^/L)	HB (g/L)	PLT (×10^9^/L)	ESR (mm/h)	C4 (mg/dl)	C3 (mg/dl)
Before treatment	30	6.46 ± 3.87	120.6 ± 27.05	224.44 ± 89.76	34.36 ± 32.91	14.24 ± 6.63	58.69 ± 22.29
After treatment	30	6.87 ± 2.45	129.56 ± 17.36	243.00 ± 61.69	11.84 ± 9.19	17.79 ± 7.24	75.17 ± 14.49
*t* value		−0.657	−2.777	−1.457	4.066	−4.869	−4.357
*p* value		.002	.000	.000	.000	.000	.005

Abbreviations: ESR, erythrocyte sedimentation rate; Hb, hemoglobin; PLT, platelet count; WBC, white blood cell count.

IgG, IgM, and ESR levels were lower after treatment than at baseline, and the differences were statistically significant (*p* < .05; see Table 2).

**Table 2:** The changes of indicators before and after treatment in the patients

	*n*	IgG (mg/dl)	IgM (mg/dl)	ds‐DNA (mg/dl)	A/G	SLEDAI score	CRP (mg/L)	The dose of prednisone (mg/d)
Before treatment	30	1493.88 ± 759.69	95.93 ± 46.61	217.38 ± 92.17	1.45 ± 0.37	18.24 + 4.1	11.82 ± 23.76	38.75 ± 4.25
After treatment	30	1029.98 ± 360.94	72.54 ± 39.19	33.75 ± 45.98	1.85 ± 0.43	4.92 + 2.9	3.21 ± 4.15	10.75 ± 34.25
*t* value		3.585	3.992	9.442	−5.62	2.241	1.839	1.245
*p* value		.008	.000	.516	.001	.015	.421	.005

Abbreviations: CRP, C‐reactive protein; IgG, immunoglobulin G; IgM, immunoglobulin M; SLEDAI, SLE Disease Activity Index.

### 3.2 Glucocorticoid doses at baseline and after treatment

Participants' doses of glucocorticoids were lower after treatment than at baseline, and this difference was statistically significant (*p* < .05; see Table 2).

### 3.3 SLEDAI‐2 K scores at baseline and after treatment

SLEDAI‐2 K scores were lower after treatment compared with baseline, and the difference was statistically significant (*p* < .05; see Table 2).

### 3.4 Urine protein test results at baseline and after treatment

The 24‐h urine protein test results in 12 cases averaged 1.54 ± 2.09 g at baseline and 0.78 ± 1.58 g after treatment. The difference was statistically significant, with *p* = .001 and *t* = 2.18.

### 3.5 Therapeutic effectiveness

The number of cases with obvious effect, effect, and no effect of treatment were 5, 23, and 2, respectively. The total effectiveness was 93.3%.

### 3.6 Adverse reactions among the five groups of patients

After treatment, two patients experienced infections, one a cytomegalovirus infection, and one a urinary tract infection. The total incidence of infection was 6.67%. No serious adverse events, infusion reactions, or hypersensitivities occurred during the treatment.

## 4 DISCUSSION

SLE is a relatively common chronic autoimmune disease. The short‐term therapeutic goals are to control disease activity, improve clinical symptoms, and achieve clinical remission, or a low level of disease activity. The long‐term therapeutic goals are to prevent and reduce relapse, reduce adverse drug reactions, prevent and control organ damage caused by the disease, and achieve long‐term sustained remission, thereby reducing the mortality rate and improving the quality of life for patients. Standard therapy has poor efficacy and a high incidence of side effects for some patients. Multiple research studies have confirmed that the addition of belimumab in SLE treatment may reduce the occurrence of adverse reactions to standard medications and delay disease progression, with a good safety profile.^7^ Belimumab was approved for marketing in China in 2019 and included in the 2020 SLE Treatment Guidelines for China.^1^

B cells in patients with SLE are highly activated in response to autoantigens, resulting in the production of a large number of autoantibodies, which triggers a series of inflammatory reactions.^8^ B‐cell‐activating factor (BAFF) is a secreted cytokine in the tumor necrosis factor family that stimulates the proliferation and differentiation of B lymphocytes in the body and is of great importance for the maturation and survival of B cells.^9^ Belimumab is a BAFF inhibitor; it specifically binds to BAFF in the serum and inhibits B‐cell maturation and activation by preventing BAFF from binding to B‐cell surface receptor 3. This leads to the inactivation of B cells in response to natural immune stimulants and promotes massive apoptosis of autoantibody‐producing B lymphocytes, thereby reducing the number of autoantibodies and exerting therapeutic effect.^10^ Wang et al.^11^ in a summary study of 3236 patients with SLE who were treated with belimumab, concluded that the drug significantly improved the clinical symptoms of skin rashes and joint and muscle pains, reduced the levels of serological indicators and SLE disease activity, and delayed the deterioration of vital organs including the kidneys, the liver, and the circulatory and central nervous systems. This is consistent with the results of the current study.

Another study found that the application of belimumab in patients previously medicated with glucocorticoids showed better clinical outcomes in those patients with active SLE, positive dsDNA, and decreased levels of C3 and C4.^12^ The results of a randomized clinical trial^13^ suggested that SLE Responder Index scores leveled off after 180 days of belimumab treatment; thus, more than 180 days of application were required to ensure efficacy. Therefore, in the current study, the observation duration of treatment was initially set at 6 months, and it was observed that after 6 months of treatment, the levels of complement (C) and immunoglobulin, and the SLEDAI‐2 K score were all significantly improved compared with baseline.

Another study reported that, after 53.3 months of treatment with belimumab, 9.7% of patients had a negative conversion of serum antinuclear antibody (ANA), which was then associated with a reduced risk of future SLE recurrence.^14^ In the current study, a decrease in ANA titer after treatment was observed only in two patients. This was possibly related to the short belimumab treatment duration.

Immune‐related anemia in SLE may be correlated with increased autoantibody‐mediated erythrocyte destruction, and a significant improvement in immune‐related anemia in patients has been reported following belimumab treatment.^15^ This is consistent with our findings. The improvement in immune‐related anemia might be related to a decrease in pathogenic antibodies with belimumab treatment, but the exact mechanism needs to be studied further.

A low A/G ratio may be a potential predictor of the development of LN in Chinese patients with SLE.^16^ The current study found a significant increase in patients' A/G ratio after treatment, which might suggest that belimumab could reduce future risk of developing LN.

Viral infection of the upper respiratory tract, diarrhea, leukopenia, and hypoglobulinemia have been reported as common adverse reactions to belimumab.^17^ Viral infection detected during the follow‐up, especially Epstein‐Barr virus infection, might easily lead to increased disease activity. In the current study, one patient developed infection and fever, and the relevant tests showed positive for cytomegalovirus antibodies. The patient was cured of the virus after antiviral treatment. One other patient developed a urinary tract infection; they were cured of the infection after symptomatic anti‐infective treatment.

## 5 CONCLUSION

In conclusion, the current study provides preliminary evidence that the levels of C, ESR, and Hb, along with SLEDAI‐2K scores and urine protein test results, improve with the addition of belimumab to standard treatment in patients recently diagnosed with SLE. This could better reduce disease activity and recurrence. In particular, patients recently diagnosed with SLE with high disease activity might see additional clinical benefits from early administration of belimumab

However, as a single‐center retrospective study, there was some bias in the selection of participants. Also, the limited number of patients, lack of a control group, and limited follow‐up duration might have contributed to skew in the data. The safety and efficacy of belimumab in Chinese patients recently diagnosed with SLE still needs to be investigated in large clinical trials.

## Data Availability

The data that support the findings of this study are available from the corresponding author, upon reasonable request.

## References

[1] Rheumatology branch of Chinese medical association, national clinical research center for skin and immune diseases, China SLE research collaborative group. 2020 Chinese guidelines for the diagnosis and treatment of systemic lupus erythematosus. Zhong Hua Nei Ke Za Zhi. 2020;59(3):172‐185(Chinese). 10.3760/cma.j.issn.0578-1426.2020.03.002 32146743

[2] He CM , Zhang FC . Belimumab: A new targeted drug for systemic lupus erythematosus. Union Medical Journal. 2020;11(2):130‐134(Chinese).

[3] Yang HX , Zhang FC . New biologic targets for systemic lupus erythematosus: hope and challenge. Union Medical Journal. 2020;11(3):241‐246(Chinese).

[4] Reátegui‐Sokolova C , Rodríguez‐Bellido Z , Gamboa‐Cárdenas RV , et al. Remission and low disease activity state prevent hospitalizations in systemic lupus erythematosus patients. Lupus. 2019;28(11):1344‐1349. 10.1177/0961203319876998 31551028

[5] Gatto M , Saccon F , Zen M , et al. Early disease and low baseline damage as predictors of response to belimumab in patients with systemic lupus erythematosus in a real‐life setting. Arthritis Rheumatol. 2020;72(8):1314‐1324. 10.1002/art.41253 32275125

[6] Aringer M , Costenbader K , Daikh D , et al. 2019 european league against Rheumatism/American college of rheumatology classification criteria for systemic lupus erythematosus. Arthritis Rheumatol. 2019;71(9):1400‐1412. 10.1002/art.40930 31385462 PMC6827566

[7] Wallace DJ , Ginzler EM , Merrill JT , et al. Heath A. safety and efficacy of belimumab plus standard therapy for up to thirteen years in patients with systemic lupus erythematosus. Arthritis Rheumatol. 2019;71(7):1125‐1134. 10.1002/art.40861 30771238 PMC6617785

[8] Blair HA , Duggan ST . Belimumab: a review in systemic lupus erythematosus. Drugs. 2018;78(3):355‐366. 10.1007/s40265-018-0872-z 29396833

[9] Shi Z , Gao CL , Xia ZK , Sun T , Zhang P . Progress in the treatment of systemic lupus erythematosus in children with belizumab. Chinese J Integ Dermatol Venereol. 2021;20(4):413‐416(Chinese).

[10] Reátegui‐Sokolova C , Rodrtguez‐Bellido Z , Gamboa‐Cárdenas RV , et al. Remission and low disease activity state prevent hospitalizations in systemic lupus erythematosus patients[j]. Lupus. 2019;8(11):1344‐1349. 10.1177/0961203319876998.PMID:31551028 31551028

[11] Wang J , Wang K , Niu CQ . System analysis of the efficacy and safety of belimumab in the treatment of systemic lupus erythematosus. J Med Inform. 2021;34(21):65‐68. 10.3969/j.issn.1006-1959.2021Nov.21.016(Chinese).

[12] Mucke J , Brinks R , Fischer‐Betz R , et al. Patient satisfaction and disease control in patients with systemic lupus erythematosus is not affected by switching from intravenous belimumab to subcutaneous injections. Patient Prefer Adherence. 2019;13:1889‐1894. 10.2147/PPA.S227208 31806937 PMC6839583

[13] Zhang F , Bae SC , Bass D , et al. A pivotal phase III, randomised, placebo‐controlled study of belimumab in patients with systemic lupus erythematosus located in China, Japan and South Korea. Ann Rheum Dis. 2018;77(3):355‐363. 10.1136/annrheumdis-2017-211631 29295825 PMC5867402

[14] Kwon OC , Kim YG , Park JH , Park MC . Seroconversion to antinuclear antibody negativity and its association with disease flare in patients with systemic lupus erythematosus. Lupus. 2020;29(7):697‐704. 10.1177/0961203320917748 32279583

[15] Wang JR , Wu X , Dilinegal E , et al. Efficacy and safety of belimumab in the treatment of systemic lupus erythematosus. Chinese J Rheumatol. 2021;25(8):529‐532(Chinese).

[16] Liu XR , Qi YY , Zhao YF , Cui Y , Wang XY , Zhao ZZ . Albumin‐to‐globulin ratio (AGR) as a potential marker of predicting lupus nephritis in Chinese patients with systemic lupus erythematosus. Lupus. 2021;30(3):412‐420. 10.1177/0961203320981139 33407045

[17] Huang X , Shi Y . A review on the treatment of systemic lupus erythematosus in children. Chong Qing Medicine. 2021;10(50):3385‐3388(Chinese).

